# Ecological shifts in avian influenza viruses following HPAIV introductions in southwestern Alaska, 2011–2024

**DOI:** 10.1038/s44298-026-00212-6

**Published:** 2026-07-17

**Authors:** Christina A. Ahlstrom, Laura C. Scott, Andrew B. Reeves, Alison R. Williams, Rebecca L. Poulson, David E. Stallknecht, Andrew M. Ramey

**Affiliations:** 1 US Geological Survey Alaska Science Center, Anchorage, AK USA; 2 US Geological Survey National Wildlife Health Center, Madison, WI USA; 3 US Fish and Wildlife Service, Izembek National Wildlife Refuge, Cold Bay, AK USA; 4 University of Georgia, Athens, GA USA

**Keywords:** Ecology, Ecology, Microbiology

## Abstract

Southwestern Alaska wetlands are critical staging habitats for migratory waterfowl that breed in western North America and East Asia. Since 2011, samples originating from hunter-harvested waterfowl and environmental feces have been collected annually in and around Izembek National Wildlife Refuge, Alaska to assess the occurrence, evolution, and spread of avian influenza viruses (AIVs). Viruses from wild birds inhabiting this region have consistently been detected, and frequent intercontinental viral dispersal has been observed. In 2014 and 2022, H5 subtype highly pathogenic avian influenza viruses (HPAIVs) were detected in the Pacific Flyway of North America, which may have been influenced by the genetic and antigenic diversity of low pathogenicity avian influenza viruses (LPAIVs) circulating among migratory birds at the time. Conversely, the introduction of HPAIVs may have altered the ecology of co-circulating LPAIVs among wild aquatic bird hosts. To assess these ecological interactions, we examined long-term patterns in LPAIV and HPAIV occurrence and diversity, focusing on changes associated with HPAI introduction events. During 14 years of surveillance for AIVs, virus prevalence peaked during periods of HPAIV introduction. Reassortment between HPAIVs and LPAIVs generated novel HPAIV genotypes and one LPAIV incorporating a HPAIV gene segment. The proportion of Eurasian-lineage gene segments increased following introduction of HPAIVs in 2022. This long-term dataset highlights the changing ecology of LPAIVs and HPAIVs among wild birds at one of North America’s most consistently sampled active surveillance sites for AIVs.

## Introduction

Migratory waterfowl, gulls, and shorebirds are the primary natural reservoirs for low pathogenic avian influenza viruses (LPAIVs)^1^. Such viruses are sporadically introduced into domestic poultry production systems where those of the hemagglutinin H5 and H7 subtypes may develop a highly pathogenic phenotype^2,3^. One particular genetic lineage of H5 subtype highly pathogenic avian influenza viruses (HPAIVs) emerged in domestic geese in Guangdong, China circa 1996^4^ and repeatedly spilled back into wild birds during 2002‒2013, affecting animals in countries throughout Europe, Asia, and Africa^5^. Since 2014, HPAIVs of the clade 2.3.4.4 sub-lineage have become established in wild bird populations^6^ and spread to North America, South America, and Antarctica^7–9^.

In 2011, researchers at the U.S. Geological Survey Alaska Science Center began partnering with the U.S. Fish and Wildlife Service to conduct annual sampling of autumn-staging waterfowl and gulls in and around Izembek National Wildlife Refuge (NWR) in southwestern Alaska to better understand avian influenza virus (AIV) ecology and to detect HPAIVs entering North America via Beringia^10^. This effort has continued through 2024^11^. Large numbers of waterfowl, including species exhibiting intercontinental migratory connectivity with Asia, such as northern pintails (*Anas acuta*) and emperor geese (*Anser canagicus*), use Izembek NWR as a staging area during their autumn migrations^12,13^. As such, this region is popular with sport and subsistence hunters, offering opportunities to sample hunter-harvested birds for AIVs. Results from previous annual sampling efforts have demonstrated a relatively high level of intercontinental viral dispersal and differences in virus prevalence and diversity among species and functional groups (i.e., dabbling ducks, diving ducks, geese)^14^.

Since consistent autumn surveillance for AIVs began in southwestern Alaska, HPAIVs have been introduced into North America by wild birds. In late 2014, H5N8 HPAIVs were introduced into North America via Beringia, which led to the emergence and spread of reassortant H5N2 viruses in the Pacific Flyway^15–18^. In late 2021 and 2022, at least four additional introductions of H5N1 HPAIVs were reported in North America, from Europe via overlapping flyways in the North Atlantic, and again from East Asia via the overlapping flyways in the North Pacific^9,19^. In 2022, HPAIVs were isolated for the first time from samples collected in and around Izembek NWR^19^.

Although previous studies have examined the evolution of HPAIVs during epidemic waves over time at a continental scale (e.g.,^20,21^), the role of LPAIV and HPAIV interactions in shaping the long-term evolution of AIVs among wild birds remains poorly understood^22^. In this study, we assessed how the ecology of AIVs changed throughout a 14-year period at Izembek NWR by analyzing data collected before, concurrent with, and after introductions of HPAIVs. Specifically, we compared annual differences in AIV prevalence, timing of detections, and subtype composition. We also investigated whether there was evidence for reassortment between LPAIVs and HPAIVs circulating among wild birds at Izembek NWR. This study elucidates long-term epidemiological patterns of AIVs in wild birds at an important site for detecting intercontinental HPAIV dispersal and novel reassortant genotypes, which may help to inform surveillance strategies and to anticipate future shifts in viral ecology.

## Results

### Avian influenza virus prevalence

A total of 13,014 samples from 27 wild bird species were collected between 2011 and 2024 in and around Izembek NWR (Table 1)^11^. The number of samples collected during autumn each year ranged between 284 in 2020 and 1483 in 2021 (Table 2). Fecal samples from glaucous-winged gulls (*Larus glaucescens*) and emperor geese accounted for 5743 samples, whereas 7271 samples originated from oropharyngeal, cloacal, or a combination of oropharyngeal/cloacal (OP/CL) swabs from hunter-harvested waterfowl. Approximately 70% of samples were derived from northern pintails, glaucous-winged gulls, and emperor geese. At least one positive AIV detection was observed in 14 different wild bird species (Table 1).

**Table 1:** Summary of samples collected from different species and functional groups as well as avian influenza virus isolation results

Functional group and sample type	Species	Total	Virus isolation +	% virus isolation + (95% CI)	# HPAIV +
dabbling duck (OP/CL)		5392	394	7.31 (6.64–8.03)	72
	Green-winged teal (*Anas carolinensis*)	834	87	10.43 (8.53–12.70)	5
	American wigeon (*Mareca americana*)	251	3	1.20 (0.24–3.62)	2
	Eurasian wigeon (*Mareca penelope*)	50	0	0 (0–8.52)	0
	Gadwall (*Mareca strepera*)	60	4	6.67 (2.16–16.39)	4
	Mallard (*Anas platyrhynchos*)	672	53	7.89 (6.07–10.19)	9
	Northern pintail (*Anas acuta*)	3457	239	6.91 (6.11–7.81)	51
	Northern shoveler (*Anas clypeata*)	68	8	11.76 (5.83–21.79)	1
diving or sea duck (OP/CL)		331	6	1.81 (0.74–3.98)	0
	Black scoter (*Melanitta americana*)	12	0	0 (0–28.20)	0
	Bufflehead (*Bucephala albeola*)	35	3	8.57 (2.21–23.13)	0
	Canvasback (*Aythya valisineria*)	1	0	0 (0–83.25)	0
	Common eider (*Somateria mollissima*)	22	0	0 (0–17.55)	0
	Common goldeneye (*Bucephala clangula*)	8	0	0 (0–37.22)	0
	Common merganser (*Mergus merganser*)	1	1	100 (16.75–100)	0
	Greater scaup (*Aythya marila*)	105	0	0 (0–4.24)	0
	Harlequin duck (*Histrionicus histrionicus*)	88	1	1.14 (0–6.77)	0
	King eider (*Somateria spectabilis*)	5	1	20.00 (2.03–64.04)	0
	Lesser scaup (*Aythya affinis*)	3	0	0 (0–61.75)	0
	Long-tailed duck (*Clangula hyemalis*)	16	0	0 (0–22.69)	0
	Red-breasted merganser (*Mergus serrator*)	14	0	0 (0–25.15)	0
	Ring-necked duck (*Aythya collaris*)	1	0	0 (0–83.25)	0
	White-winged scoter (*Melanitta deglandi*)	20	0	0 (0–18.98)	0
goose (OP/CL)		1543	7	0.45 (0.20–0.95)	4
	Black brant (*Branta bernicla nigricans*)	875	1	0.11 (0–0.71)	0
	Cackling goose (*Branta hutchinsii*)	612	4	0.65 (0.19–1.73)	4
	Emperor goose (*Anser canagicus*)	51	2	3.92 (0.33–13.97)	0
	Greater white-fronted goose (*Anser albifrons*)	5	0	0 (48.91)	0
gull (OP/CL)	Glaucous-winged gull (*Larus glaucescens*)	3	0	0 (0–61.75)	0
other (OP/CL)	Sandhill crane (*Antigone canadensis*)	1	0	0 (0–83.25)	0
goose (F)	Emperor goose	2817	38	1.35 (0.98–1.85)	0
gull (F)	Glaucous-winged gull	2926	35	1.20 (0.86–1.66)	0

Abbreviations used within the table are as follows: *OP/CL* oropharyngeal and/or cloacal swab, *F* fecal swab, *CI* confidence interval, *HPAIV* highly pathogenic avian influenza virus.

**Table 2:** Summary of wild bird samples collected each year (2011–2024) in southwestern Alaska and avian influenza virus isolation (VI) results from oropharyngeal and/or cloacal swabs (OP/CL) collected from hunter-harvested ducks and geese as well as from fecal samples (F) collected from live emperor geese (EMGO) and glaucous-winged gulls (GWGU)

Year	Total samples collected	Duck/goose^a^ (OP/CL)	Duck/goose %VI + (95% CI)	EMGO (F)	EMGO %VI + (95% CI)	GWGU (F)	GWGU %VI + (95% CI)	% HPAI + (# detections)
2011	995	744	4.17 (2.93–5.87)	99	2.02 (0.11–7.51)	152	1.97 (0.41–5.90)	0
2012	979	379	5.80 (3.82–8.67)	298	1.01 (0.20–3.06)	302	0.33 (0–2.04)	0
2013	944	428	3.50 (2.09–5.75)	263	0.38 (0–2.34)	253	4.74 (2.65–8.19)	0
2014	987	350	9.71 (7.00–13.30)	292	1.03 (0.21–3.12)	345	0.87 (0.17–2.65)	0
2015	937	527	6.83 (4.95–9.33)	207	1.93 (0.58–5.04)	203	0.49 (0–3.02)	0
2016	947	546	6.04 (4.31–8.39)	183	1.64 (0.34–4.93)	218	0.46 (0–2.80)	0
2017	642	333	5.41 (3.39–8.43)	134	0 (0–3.35)	175	0.57 (0–3.49)	0
2018	823	318	4.72 (2.82–7.70)	253	2.37 (0.97–5.20)	252	0.40 (0–2.44)	0
2019	981	400	4.25 (2.62–6.75)	300	0.67 (0.02–2.56)	281	1.42 (0.42–3.74)	0
2020	284	263	4.94 (2.83–8.35)	12	0 (0–28.20)	9	0 (0–34.46)	0
2021	1483	566	3.89 (2.55–5.84)	473	2.11 (1.10–3.90)	444	1.35 (0.55–2.99)	0
2022	1008	811	10.60 (8.66–12.92)	105	1.90 (0.10–7.10)	92	1.09 (0–6.36)	5.36% (54)
2023	1001	861	2.79 (1.86–4.13)	75	0 (0–5.84)	65	1.54 (0–9.00)	0.40% (4)
2024	1000	742	5.53 (4.08–7.43)	123	1.63 (0.08–6.10)	135	0 (0–3.33)	1.80% (18)

^a^Refer to Table 1 for all species included in duck/goose.

The overall prevalence of AIVs in OP/CL swabs ranged from 0.5% (95% confidence interval (CI): 0.2–1.0) in geese to 7.3% (95% CI: 6.6–8.0%) in dabbling ducks (Table 1). In fecal swabs, AIV prevalence was 1.2% (95% CI: 0.9–1.7%) and 1.4% (95% CI: 1.0–1.9%) in glaucous-winged gulls and emperor geese, respectively. Diving or sea ducks represented a minority of the total number of samples collected (*n* = 331, 2.6%) and had an overall prevalence of 1.8% (95% CI: 0.7–4.0%). Although several species of diving or sea ducks had a relatively high prevalence of AIVs, the uncertainty around estimates was high given their relatively small (*n* < 50) samples sizes (Table 1).

**Fig. 1:**
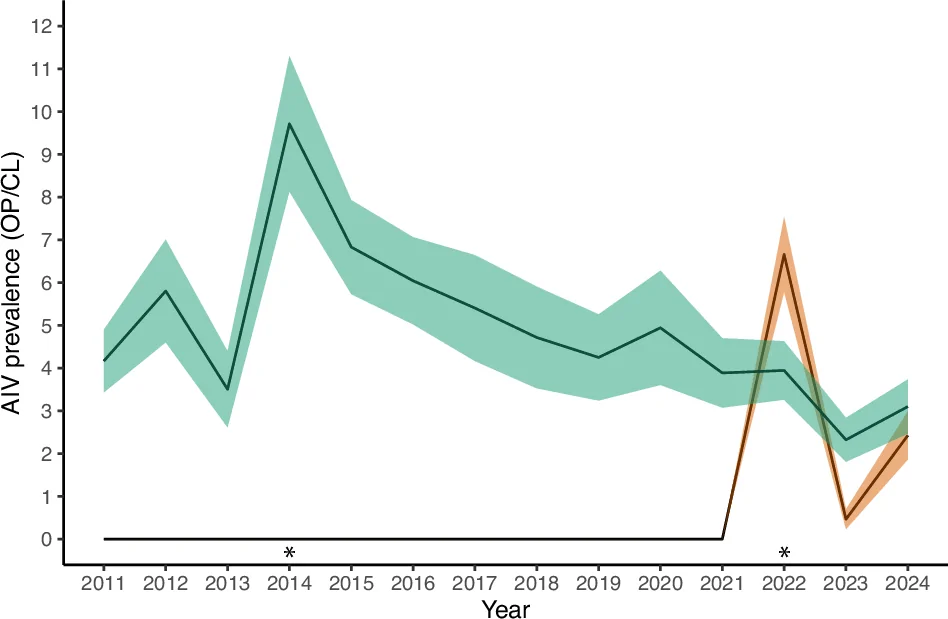
Annual prevalence of low pathogenicity avian influenza (green) and highly pathogenic avian influenza (HPAI; orange) viruses in oropharyngeal/cloacal swabs from ducks and geese collected in southwestern Alaska between 2011 and 2024. Shading represents standard error and asterisks (*) indicate the initial detections of HPAI viruses in the Pacific Flyway in 2014 and 2022.

AIVs from OP/CL swabs were detected every year from ducks and geese, but prevalence differed between years, ranging between 2.8% (95% CI: 1.9–4.1%) in 2023 to 10.6% (95% CI: 8.7–12.9%) in 2022 (Table 2). A generally decreasing trend in LPAIV prevalence was also observed after a peak prevalence in 2014 (Fig. 1). In fecal samples from gulls and emperor geese, prevalence of AIVs was generally lower than OP/CL samples from ducks and geese (Tables 1 and 2). The highest number of AIV isolates were obtained from green-winged teal (*Anas crecca*; *n* = 87), emperor geese (*n* = 40), glaucous-winged gulls (*n* = 35), mallards (*A. platyrhynchos*; *n* = 53), and northern pintails (*n* = 239). The percentage of samples collected annually from these five species ranged between 54.5% in 2011 and 97.2% in 2019 (Supplementary Fig. 1).

We detected HPAIVs for the first time in 2022 (Table 2 and Fig. 1). A total of 76 HPAIVs were detected among seven species of ducks and geese during 2022–2024, all from OP/CL swabs, with most (*n* = 51) from northern pintail ducks (Table 1). HPAIV prevalence in all samples was higher in 2022 (5.4%, 95% CI: 4.1–6.9%) than in 2023 (0.4%, 95% CI: 0.1–1.1%) and 2024 (1.8%, 95% CI: 1.1–2.8%). In 2022, the prevalence of HPAIVs in OP/CL swabs was higher (6.3%, 95% CI: 4.8–8.1%) than LPAIVs (3.7%, 95% CI: 2.6–5.2%; Fig. 1).

**Fig. 2:**
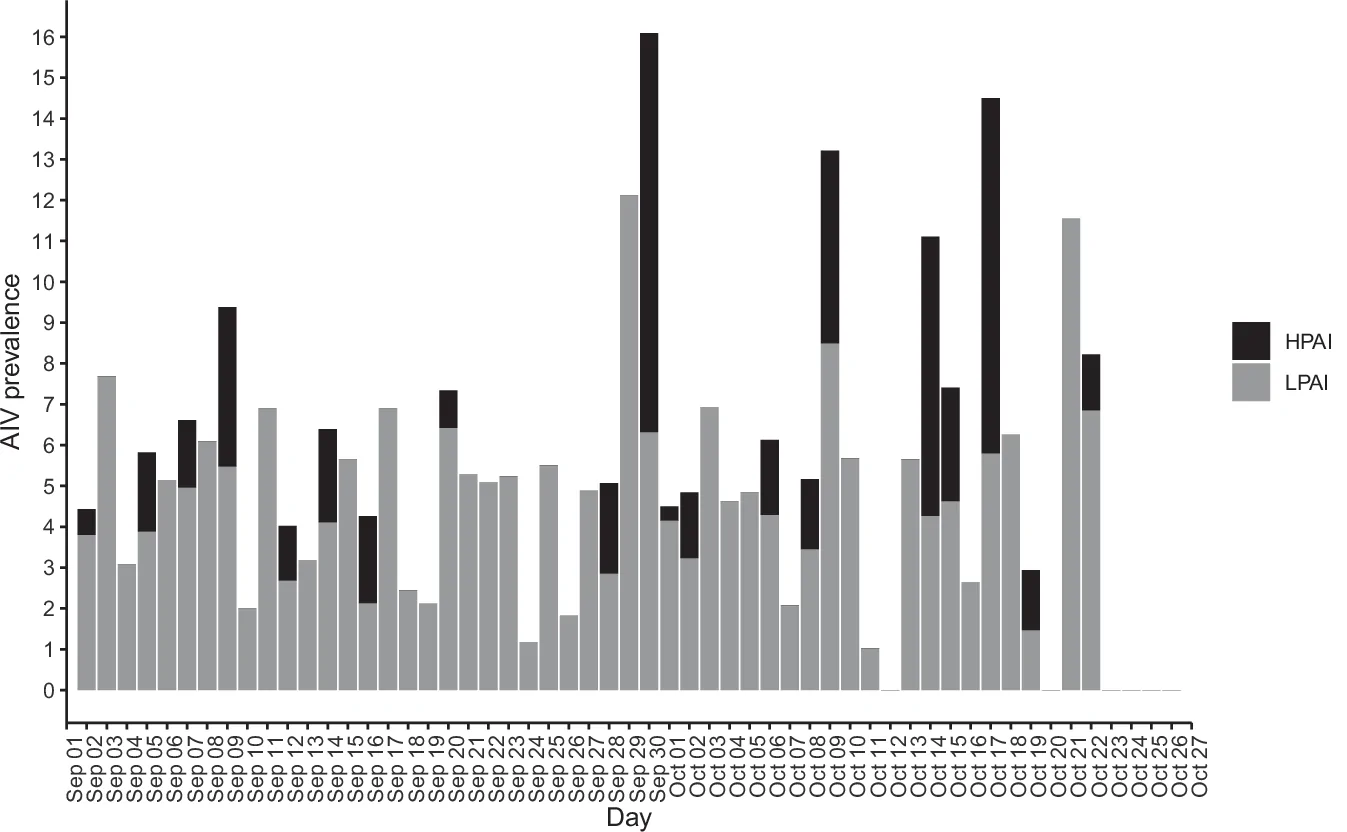
Daily avian influenza virus prevalence (%) in oropharyngeal/cloacal swabs from ducks and geese collected during autumn in southwestern Alaska during 2011–2024. Shading indicates viral pathogenicity: low pathogenicity avian influenza (LPAI) = light gray, highly pathogenic avian influenza (HPAI) = dark gray.

We calculated the daily prevalence of AIVs in OP/CL samples across all years combined. Both LPAIVs and HPAIVs were detected throughout the two-month autumn sampling period, with daily AIV prevalence ranging between 0 and 16.1% (Fig. 2). No obvious trends in average daily prevalence were identified throughout the two-month period, though higher peaks of prevalence were observed in late September through October.

### Avian influenza virus subtypes and HPAI virus genotypes

**Fig. 3:**
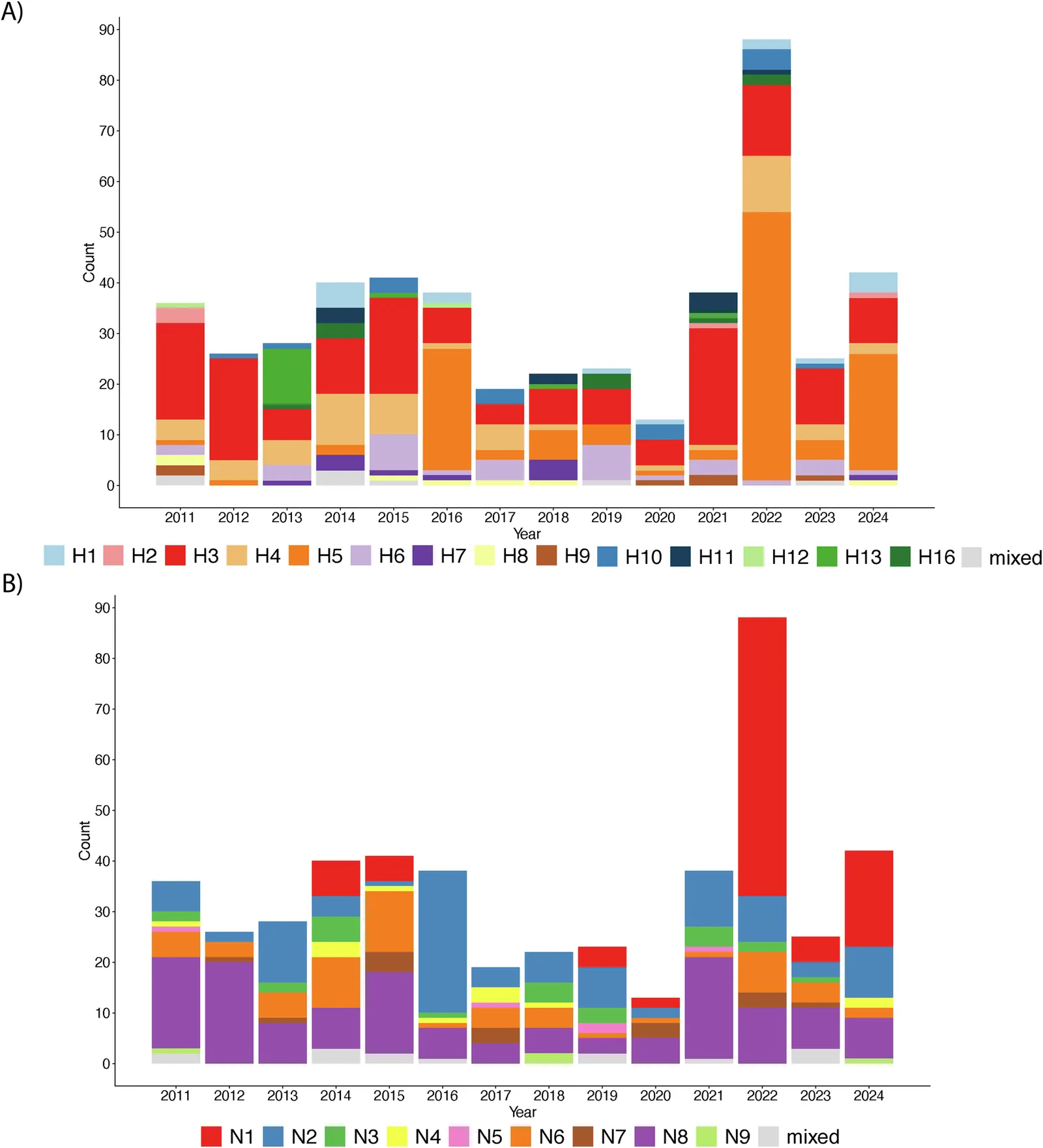
Avian influenza virus subtypes detected each year during 2011–2024. Bar chart indicating hemagglutinin (**A**) and neuraminidase (**B**) subtypes detected in wild birds from southwestern Alaska. Colors indicate different subtypes.

Complete or partial AIV genomes were obtained from 480 isolates. With the exceptions of H14 and H15, all other avian hemagglutinin (HA) and neuraminidase (NA) subtypes were detected (Fig. 3). A total of 40 subtype combinations were detected, the most common of which were H3N8 (*n* = 121), H5N1 (*n* = 75), H5N2 (*n* = 45), and H4N6 (*n* = 44) (Supplementary Table 1). H3N8 was the only subtype detected in all 14 years. Differences in the relative occurrence of HA and NA subtype pairings were observed. For example, N2 was paired with the highest number of HA subtypes (*n* = 10) and no HxN2 subtype combination represented >50% of isolates (Table 3). In contrast, N8 was almost exclusively paired with H3 (94% of pairings) with few isolates (*n* = 8) representing other HxN8 combinations (Table 3). The proportion of three of the four most common viral subtypes differed significantly (*p* < 0.05) between the periods prior to and following the detection of HPAI southwest Alaska. H3N8 and H5N2 were significantly more common during 2011–2021, whereas H5N1 was significantly more common during 2022–2024. In contrast, the proportion of H4N6 viruses did not differ significantly (*p* = 1.00) between periods.

**Table 3:** Summary of hemagglutinin (H) and neuraminidase (N) subtype combinations detected from avian influenza virus isolates originating from wild bird samples collected in southwestern Alaska during 2011–2024

Shading of boxes represents the relative occurrence of subtype pairings (dark gray = >50 detections, medium gray = 10–50 detections, light gray = <10 detections, and white = not detected).

All H5N1 viruses detected were HPAIVs as inferred from the presence of a polybasic hemagglutinin cleavage site. One HPAIV was confirmed as only H5, as the NA gene segment sequence failed quality controls. In 2022 and 2024, H5N1 was the most common combined subtype detected. In 2022, five HPAIV H5N1 genotypes were detected (A3, A4, B3.2, B4.1, and a minor genotype), two of which were comprised of fully Eurasian-lineage gene segments and were presumed introductions from East Asia based on their similarity to viruses originating from Japan. In 2023, all four HPAIVs were of the A3 H5N1 genotype and in 2024, A3 and D1.1 HPAIV H5N1 genotypes were detected.

Accounting for the number of isolates recovered from each of the five species with the highest number of AIV detections, green-winged teal hosted a higher richness of viral subtypes as compared to northern pintails as inferred from OP/CL swabs (Supplementary Fig. 3). Viral subtype richness was similar between emperor geese and glaucous-winged gulls using fecal swabs (Supplementary Fig. 3), though the proportion of individual HA and NA subtypes varied (Supplementary Fig. 2). For example, H13 represented 34% of HA viral subtypes detected among glaucous-winged gull samples, though this subtype was not detected from emperor geese or any other species (Supplementary Fig. 2A and Supplementary Table 1).

### Continental affiliation

Eurasian or North American continental affiliation was assigned to each gene segment based on phylogenetic clustering with reference sequences for 413 viral isolates with complete whole genome sequencing data. Five percent of isolates (*n* = 22) were composed of fully Eurasian-lineage gene segments and were predominately HPAI H5N1 viruses (*n* = 20). Twenty-six percent of isolates (*n* = 109) were composed of fully American-lineage gene segments. Fully Eurasian-lineage viruses were detected in 2016, 2022, 2023, and 2024 in samples collected from green-winged teal, mallards, and northern pintails. Seventy-four percent (*n* = 304) of viral isolates had at least one Eurasian-lineage gene segment. The proportion of Eurasian-lineage gene segments within each virus was significantly greater (*p* < 0.001) during the period in which HPAIVs were detected (i.e., 2022–2024) (Supplementary Fig. 4).

### Reassortment between HPAI and LPAI viruses

**Fig. 4:**
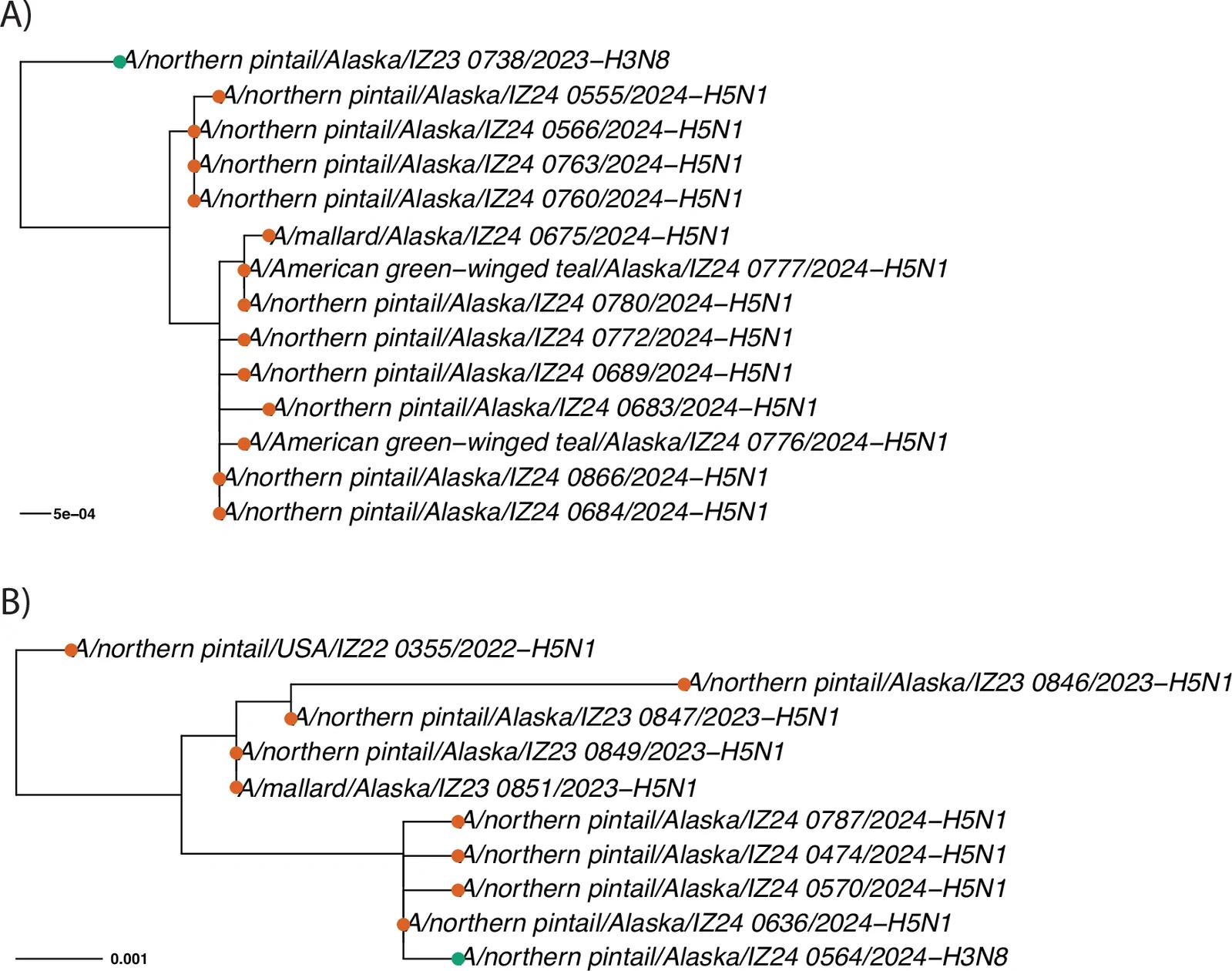
Phylogenetic clades depicting probable reassortment between low pathogenicity and high pathogenicity avian influenza viruses. Clades were extracted from maximum likelihood trees (which were created using individual gene segments) indicating probable reassortment between low pathogenicity avian influenza viruses (LPAIVs; green) and highly pathogenic avian influenza viruses (HPAIVs; orange). **A** Evidence for incorporation of descendants of the North American-lineage PB2 gene segment from a H3N8 LPAIV sampled in 2023 into H5N1 HPAIVs sampled in 2024. **B** Evidence for incorporation of descendants of the Eurasian-lineage NP gene segment from a H5N1 HPAIV into a H3N8 LPAIV. Complete phylogenetic trees for each gene segment can be found in Supplementary Fig. 5.

Phylogenetic analysis and pairwise identity comparisons of viral sequences for each gene segment provided evidence for reassortment between HPAIVs and LPAIVs (Fig. 4). Potential reassortment supported by phylogenies and greater than 99% nucleotide identity between HPAIV and LPAIV gene segments was observed for PB2, PA, NP, and NA gene segments. Eight distinct phylogenetic clades for these gene segments were found to contain both LPAIV and HPAIV sequences sharing >99% nucleotide identity (Supplementary Fig. 5). HPAIV genotypes sharing >99% nucleotide identity to LPAIV gene segments included D1.1 (four gene segments), the minor genotype (three gene segments), A3 (one gene segment), and B4.1 (one gene segment) (Supplementary Fig. 5). Reassortment appeared to occur in both directions (i.e., LPAIVs donating gene segments to HPAIVs and vice versa) based on positions of sequences in phylogenies and year of sample collection (Fig. 4). For example, the PB2 gene from an LPAIV recovered in 2023, A/northern pintail/Alaska/IZ23_0738/2023(H3N8), was basal to and shared 99.52% nucleotide identity with a HPAIV recovered in 2024, A/northern pintail/Alaska/IZ24_0760/2024(H5N1) (Fig. 4A). Conversely, the NP gene from HPAIVs sampled in 2022, 2023, and 2024 were basal to and shared up to 99.78% similarity to an LPAIV sampled in 2024, A/northern pintail/Alaska/IZ24_0564/2024(H3N8) (Fig. 4B).

## Discussion

By assessing prevalence, timing of detections, subtype diversity, and reassortment of AIVs among wild birds inhabiting southwestern Alaska over 14 years, we gained important insights into the ecology and intersections of HPAIVs and LPAIVs. Despite multiple introductions of HPAIVs into wild birds, overall AIV prevalence remained within a consistent range using hunter-harvested duck and goose samples (3.5–10.6%) and environmentally collected feces from emperor geese (0–2.4%) and glaucous-winged gulls (0–4.7%). Initial HPAIV detections in the Pacific Flyway (2014 and 2022) coincided with peaks in AIV prevalence in and around Izembek NWR, which may be related to population immunity. Subtype composition shifted following HPAIV detection in southwestern Alaska in 2022, likely driven by a predominance of H5N1 subtype viruses. Reassortment was observed between HPAIVs and LPAIVs and most AIV genome constellations contained gene segments of both Eurasian and North American lineages.

### Avian influenza virus prevalence

LPAIVs were isolated from ducks and geese in and around Izembek NWR during all 14 years of annual autumn sample collection. The highest prevalence of LPAIVs in OP/CL swabs occurred in 2014, the same year as the first wild bird-mediated incursion of HPAIV into the Pacific Flyway of North America was documented^23^ (though no HPAIVs were identified in our surveillance efforts in southwestern Alaska that year). The highest overall prevalence of AIV (LPAIVs and HPAIVs combined) was in 2022, the same year additional HPAIV introductions were identified in the Pacific Flyway of North America^9^. It is unclear why the two highest peaks in AIV prevalence among wild birds in southwestern Alaska were generally coincident with introductions of HPAIV, but it is possible that ebbs in population immunity, driven by relatively lower LPAIV prevalence in the years prior (i.e., 2013 and 2021), facilitated viral incursions and potentially also establishment among susceptible birds.

OP/CL samples from dabbling ducks yielded the highest number of AIV detections, as well as the most HPAIV isolates, consistent with previous research at this location and elsewhere^1,14^. AIV prevalence varied among years and host groups, with AIV prevalence in glaucous-winged gull feces exceeding prevalence in OP/CL swabs from dabbling ducks only once, in 2013. Sample sizes of diving or sea duck species were generally too small to confidently estimate AIV prevalence (as indicated by wide 95% confidence intervals), though lower prevalence in these species as compared to dabbling ducks has previously been observed^24,25^. No HPAIVs were detected among samples collected from diving or sea ducks, gulls, or emperor geese, suggesting that active sampling of these species may be of lower priority if the sole objective of a sampling strategy is the most efficient detection of AIVs, including HPAIVs. These results highlight where sampling efficiencies may be incorporated when resources are limited (e.g., prioritizing sampling using OP/CL swabs from dabbling ducks), but also how using long-term and varied sampling approaches can reveal trends that might otherwise be obscured by annual fluctuations, taxonomic biases, or differences in sampling methodology.

Prevalence estimates for AIVs based on hunter-harvested samples are potentially less biased than active surveillance efforts using bait trapped birds or live decoys^26,27^. Artificially increasing waterfowl density may increase AIV prevalence in hosts and their aquatic habitats and introduce bias into estimates of subtype diversity. Conversely, hunter-harvested samples are contingent on sample availability, which is generally biased towards the most commonly targeted or desirable species and temporally restricted to hunting seasons. The OP/CL swab samples from southwestern Alaska are therefore biased toward northern pintails, mallards, green-winged teal, black brant (*Branta bernicla nigricans*), and cackling geese (*Branta hutchinsii*) during the autumn staging migration period, and prevalence estimates may not represent viral occurrence in other waterfowl species or in these same taxa at other times of the year.

### Avian influenza virus subtypes and continental affiliations

Diverse AIV subtypes were observed in southwestern Alaska throughout the 14-year sampling period. A shift in the relative occurrence of three out of the four most frequently detected subtypes was observed following the detection of HPAIVs in southwestern Alaska. This was likely driven by the incursion of HPAI H5N1, which became the most frequently detected viral subtype in 2022 and 2024, and seemingly displaced H3N8 and H5N2 subtypes. Cyclical subtype dominance has previously been observed at a regional level (e.g., an inverse relationship between the relative occurrence of the H3 and H4 subtype AIVs)^28^, but we did not find evidence for this in our dataset. This could be a function of limited spatial representation in our sampling effort (e.g., local versus regional), differences in sampling methodology (e.g., hunter-harvest versus live-trapping), or unknown ecological differences. Green-winged teal seemed to harbor the highest richness of viral subtypes as inferred through rarefaction, though it remains unclear how this may relate to their life history characteristics (e.g., foraging habits, migratory tendencies^1,29^) or other factors. Less common viral subtypes were more frequently (e.g., H9 and H16) or exclusively (H13), detected in fecal samples from gulls and emperor geese, which would have been missed had we relied solely on OP/CL swabs from hunter-harvested birds. This indicates that sampling from multiple functional groups, including those that are less commonly or not targeted by hunters, can provide a more comprehensive summary of AIV diversity.

Southwestern Alaska is a staging area for birds with migratory connectivity to both North America and East Asia^30,31^. Our results are consistent with previous observations that AIVs isolated from birds inhabiting this region have a relatively high proportion of Eurasian-lineage gene segments as compared to other locations within North America^14,32^. This could be partly driven by northern pintails and emperor geese because both species have migratory connectivity to Asia, are locally abundant in southwestern Alaska during autumn, and are consistent hosts of AIVs^32–34^. A higher proportion of Eurasian-lineage gene segments among AIVs was identified following HPAIV detection, which is unsurprising given that all HPAI viral genotypes detected in and around Izembek NWR retained between four and eight Eurasian-lineage gene segments.

Out of seven H5N1 HPAIV genotypes detected statewide in Alaska in 2022^35^, five were detected in OP/CL samples collected from waterfowl in and around Izembek NWR. Two of these genotypes (A4 and a minor genotype) have not been reported in North America outside of Izembek NWR to-date. The number of confirmed HPAIV detections at Izembek NWR in 2022 accounted for 29% of HPAIV detections statewide^35^. Thus, sampling in and around Izembek NWR may capture much of the HPAIV diversity found in Alaska including variants that may not be detected elsewhere.

### Reassortment between HPAI and LPAI viruses

We found evidence of reassortment between HPAIVs and LPAIVs in southwestern Alaska following the detection of HPAIVs in 2022. Although not detected by methods used in this study, evidence for a similar reassortment between HPAIVs and LPAVs was previously reported using samples collected in southwestern Alaska in 2014^16,18^. Reassortment between viruses is an important evolutionary strategy that can enable host immune system evasion and increase viral fitness^36,37^. HPAIVs have more frequently reassorted with LPAIVs in recent years^38,39^, which may have facilitated HPAI H5N1 virus spread and establishment throughout North America as well as the generation of many novel genotypes^9^.

In samples collected during 2024, we observed one LPAIV harboring an internal gene segment (NP) apparently inherited from A3 genotype HPAIVs. The A3 genotype was the most persistent HPAIV genotype in and around Izembek NWR during 2022–2024, as it was detected in all three years. This genotype harbors an amino acid substitution N319K in the NP gene segment^40^, which has been associated with increased efficiency of viral replication^41^. Given this LPAIV reassortant was only identified in one sample, this finding may be insignificant. However, as HPAIVs continue to be maintained and evolve within a wild bird reservoir^21^, reassortment between HPAIVs and LPAIVs may increasingly affect the ecology and transmission dynamics of diverse AIVs^42^.

Four gene segments from LPAIVs that were detected in Izembek NWR seem to be potential viral ancestors to gene segments of genotype D1.1 H5N1 HPAIVs based on phylogenies and >99% similarity. Genotype D1.1 H5N1 HPAIVs have spread widely, becoming the predominant HPAIVs within North America^43^. This D1.1 genotype is a descendant of the A3 genotype and includes a North American lineage N1 gene segment that was closely related to LPAIV gene segments at Izembek NWR in 2024. Genotype D1.1 viruses have produced considerable losses in poultry and wild birds, including two fatal infections in endangered whooping cranes (*Grus americana*)^44^. Genotype D1.1HPAIVs have also infected dairy cattle and humans, in some cases fatally^45–48^. Additional research would be needed to fully understand the contribution of LPAIV-origin gene segments to HPAIV fitness and virulence; however, evidence from this study and prior investigations in southwestern Alaska^16,18,43^ indicates that HPAIV genotypes with genetic ancestry traced back to this region may have significant economic and ecological consequence at national and international scales.

Fourteen years of active surveillance for AIVs in wild birds in southwestern Alaska provides important long-term data regarding viral prevalence, subtype diversity, intercontinental dispersal, and viral evolution. This active sampling strategy was designed, in part, to detect HPAIV incursions, which was successful during 2022–2024. Continued sampling for, and characterization of, both HPAIVs and LPAIVs will further elucidate changes in the ecology of AIVs during an unprecedented epizootic. Epidemiologic patterns of prevalence and reassortment of HPAIVs in southwestern Alaska are generally consistent with those observed among endemically occurring LPAIVs that have been maintained by birds for decades and likely much longer. These shared patterns indicate that HPAIVs have the potential for long-term persistence in wild bird populations, indicating that these animal health challenges may continue.

## Methods

### Sample collection and virus detection

Wild birds, including hunter-harvested waterfowl, live glaucous-winged gulls, live emperor geese, and a single mallard found dead in 2024 suspected to be affected by HPAI, were sampled for AIVs annually during September and October of 2011–2024 within and around Izembek NWR in southwestern Alaska. Swab samples were collected from hunter-harvested birds by applying a polyester-tipped applicator to the cloaca only (2011–2014) or the cloaca and oropharynx (2015–2024) of each bird. Paired oropharyngeal and cloacal (OP/CL) samples were combined in a single cryovial with viral transport media. The collection of an oropharyngeal swab and pairing with a cloacal swab from the same hunter-harvested bird started in 2015 to optimize the probability of detection of HPAIV following an initial introduction by migratory birds into North America in 2014. Throughout the entire 2011–2024 sampling period, fecal deposits from monospecific flocks of emperor geese and glaucous-winged gulls were opportunistically swabbed with a polyester-tipped applicator following the observed flushing of birds or through identification of distinct feces. Samples were collected over the course of multiple days and at different locations to target birds inhabiting different areas. All swabs (CL, OP/CL, or fecal) were placed in cryovials with 2 mL chilled viral transport media supplemented with antibiotics. Samples were then placed in dry shippers charged with liquid nitrogen and transferred to −80 °C freezers until testing.

In the laboratory, RNA was extracted from all cloacal (2011–2014), paired OP/CL (2015–2024), and fecal (2011–2024) swab samples using the MagMax AI/NDV RNA extraction kit (Applied Biosystems, Waltham, Massachusetts) or Qiagen viral RNA mini kit (Qiagen Inc., Venlo, The Netherlands). RNA extracts were screened using real-time reverse transcriptase polymerase chain reaction (rRT-PCR) targeting the influenza A virus matrix gene^49^. For samples collected in 2011–2017, samples yielding cycle threshold (Ct) values < 45 for the AIV matrix gene were inoculated into specific pathogen-free embryonated chicken (*Gallus gallus domesticus*) eggs for virus isolation. All samples collected during 2018–2024 were inoculated into embryonated chicken eggs for virus isolation. Amnio-allantoic fluids were collected after 120 h and tested via hemagglutination assay. RNAs extracted from hemagglutination assay positive amnio-allantoic fluids were screened for the influenza A virus matrix gene and H5 clade 2.3.4.4b hemagglutinin gene (2022–2024 samples only) by rRT-PCR. Isolates that yielded a cycle threshold value < 40 in rRT-PCR targeting H5 clade 2.3.4.4b were submitted to the U.S. Department of Agriculture (USDA) National Veterinary Services laboratory (Aimes, IA) for official confirmation of pathotype and subtype. All other influenza A viral isolates were genomically sequenced using short-read sequencing (Illumina Inc., San Diego, California) and assembled to reference sequences as previously described^14,19,50^. Nucleotide sequences from samples that were genomically sequenced were submitted to the National Center for Biotechnology Information (NCBI) GenBank website and made publicly available at http://www.ncbi.nlm.nih.gov/genbank/.

### Phylogenetic analyses and bioinformatics

To assign continental affiliation, all full-length influenza A virus sequences sampled from avian species with a collection date during January 1, 2011–December 31, 2024 were downloaded from the Influenza Virus Database (https://www.ncbi.nlm.nih.gov/genomes/FLU/Database/nph-select.cgi) on August 25, 2025. Sequences originating from domestic birds were removed. For each gene segment, up to 50 sequences from each year were randomly selected and assigned continental affiliation “Eurasian,” “American,” or “other” based on sample collection location. The category “other” included AIVs sampled in Australia or New Zealand. Gene segment alignments of downloaded reference sequences and those from complete genomes from this study were generated. Neighbor-joining trees for each gene segment were estimated using the R^51^ package “phangorn”^52^. Sequences from Alaska were assigned “Eurasian” or “North American” lineage based on phylogenetic clustering with downloaded reference sequences. In the absence of sufficient references, sequences were queried using BLAST and continental affiliation was assigned based on the geographic sampling location for the top five hits^53^.

Maximum likelihood phylogenetic trees for each gene segment were estimated. Separate alignments for each gene segment were created using Geneious^54^. The package “phangorn”^52^ in R was used to generate maximum likelihood phylogenetic trees using the nucleotide substitution model with the lowest AICc and 1000 bootstrap replicates.

Reassortment between LPAI and HPAI viruses was inferred when phylogenetic clades contained both LPAI and HPAI viral sequences. Percent nucleotide identity was confirmed through the generation of distance matrices for each gene segment. We defined LPAI and HPAI viral gene segments sharing >99% nucleotide identity as indicating a likely recent reassortment event. Sequences from HPAI virus isolates were assigned genotypes using GenoFlu (v1.05^9^).

### Statistical analyses

Rarefaction curves were generated to determine subtype diversity for species from which 10 or more virus isolates were recovered. The R package “vegan”^55^ using the function “rarefy” was employed to determine the expected subtype richness for different species in random subsamples of the data. AIV prevalence and 95% confidence intervals were calculated using the package “binom”^56^ in R. Fisher’s exact tests with Bonferroni corrections were used to evaluate differences in the proportions of the four most common viral subtypes between the period prior to HPAIV detection (i.e., 2011–2021) and following HPAIV detection (i.e., 2022–2024), using the package “rstatix”^57^ in R.

## Supplementary information

Supplementary materials

## Data Availability

All viral genome sequences generated in this study are publicly available in the NCBI GenBank database. Accession numbers and associated metadata are provided in reference 11.
