# Supplementary material for: Ecological shifts in avian influenza viruses following HPAIV introductions in southwestern Alaska, 2011–2024
Source: Npj Viruses. 2026 Jul 17;4:55. doi: 10.1038/s44298-026-00212-6 (PMC13748544; doi:10.1038/s44298-026-00212-6)
Supplement: Supplementary file 1 [file 44298_2026_212_MOESM1_ESM.pdf]

Supplementary Figures and Tables

Ecological shifts in avian influenza viruses following HPAI virus introductions in southwestern Alaska, 2011–2024

Christina A Ahlstrom[1*], Laura C Scott[1], Andrew B Reeves[2], Alison R Williams[3], Rebecca L Poulson[4], David E Stallknecht[4], Andrew M Ramey[1*]

[1] US Geological Survey Alaska Science Center, Anchorage, AK, USA

[2] US Geological Survey National Wildlife Health Center, Madison, WI, USA

[3] US Fish and Wildlife Service, Izembek National Wildlife Refuge, Cold Bay, AK, USA

[4] University of Georgia, Athens, GA, USA

* Corresponding author

Email: cahlstrom@usgs.gov, aramey@usgs.gov

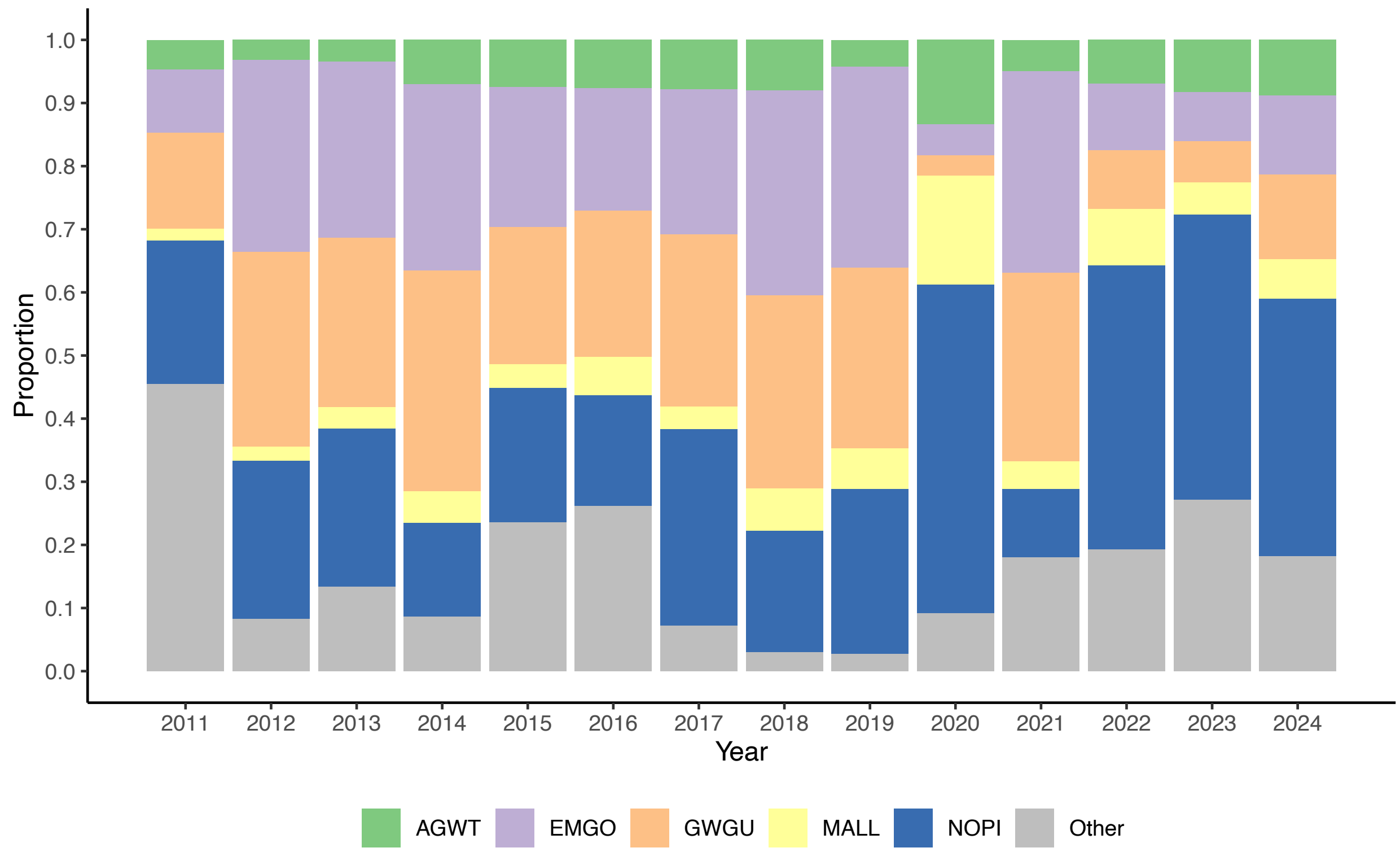

Supplementary Figure 1 Proportion of samples collected each year (2011-2024) from the five species yielding the most viruses and other wild birds sampled at Izembek NWR (AGWT = green-winged teal; EMGO = emperor goose; GWGU = glaucous-winged gull; MALL = mallard; NOPI = northern pintail).

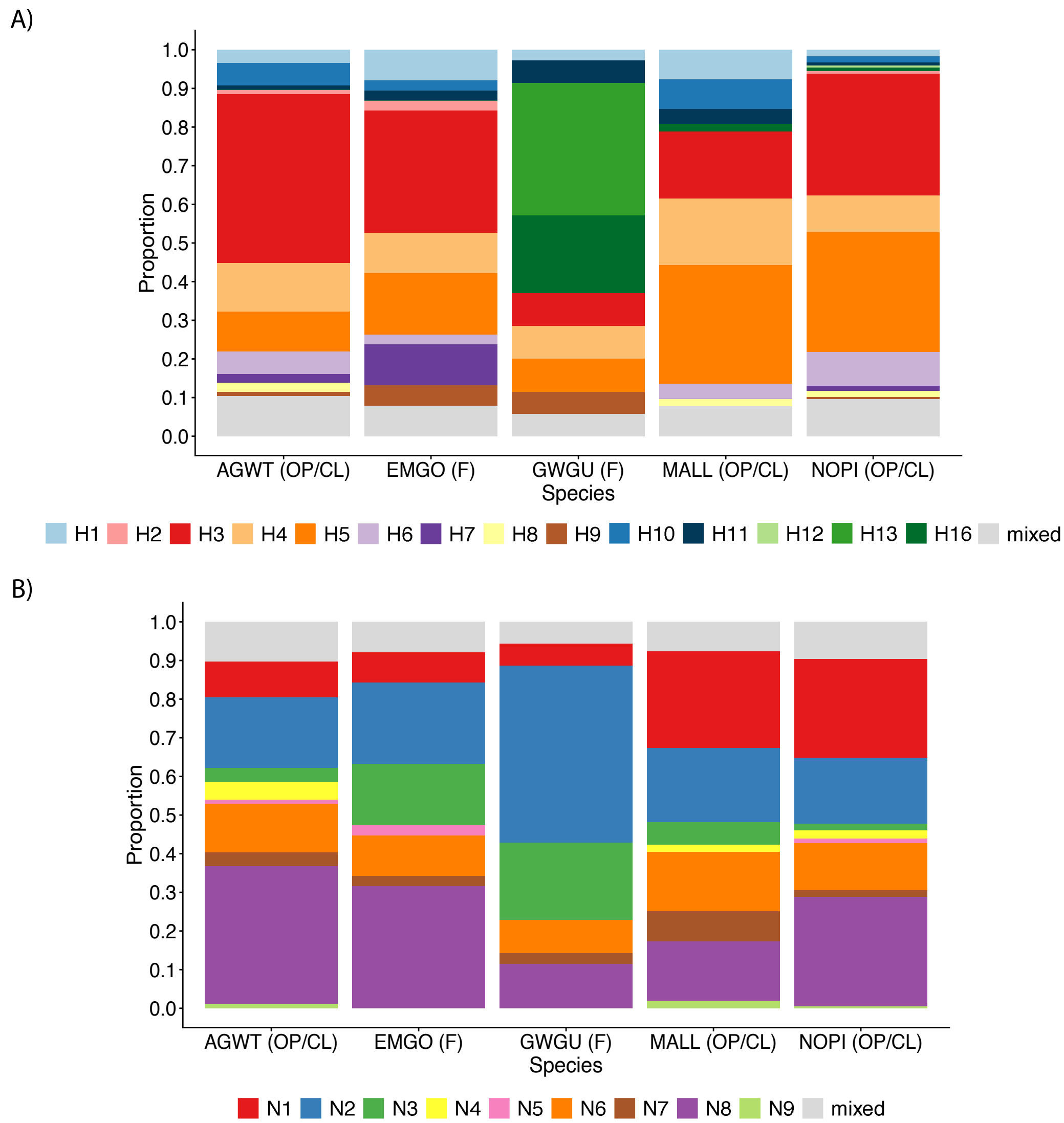

Supplementary Figure 2 Proportion of hemagglutinin (panel A) and neuraminidase (panel B) avian influenza virus subtypes detected in isolates recovered from five wild bird species sampled in southwestern Alaska during 2011-2024 ((AGWT = green-winged teal; EMGO = emperor goose; GWGU = glaucous-winged gull; MALL = mallard; NOPI = northern pintail). Sample type is indicated (OP/CL = oropharyngeal and/or cloacal swab; F = Fecal swab).

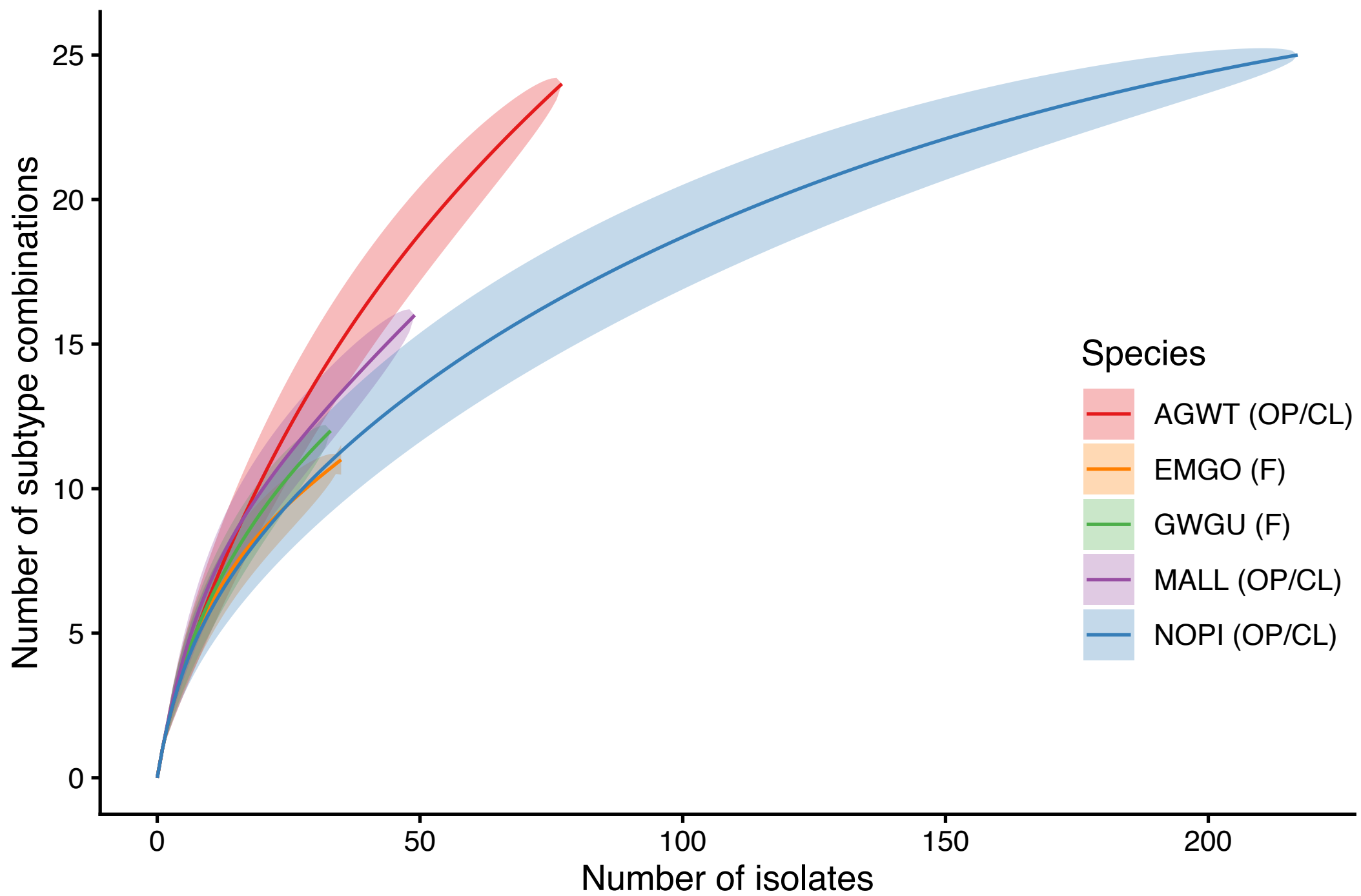

Supplementary Figure 3 Rarefaction curve depicting the expected mean avian influenza virus subtype combination richness based on the number of viruses recovered from five bird species sampled. Shaded areas represent standard error (AGWT = green-winged teal; EMGO = emperor goose; GWGU = glaucous-winged gull; MALL = mallard; NOPI = northern pintail). Sample type is indicated (OP/CL = oropharyngeal and/or cloacal swab; F = Fecal swab).

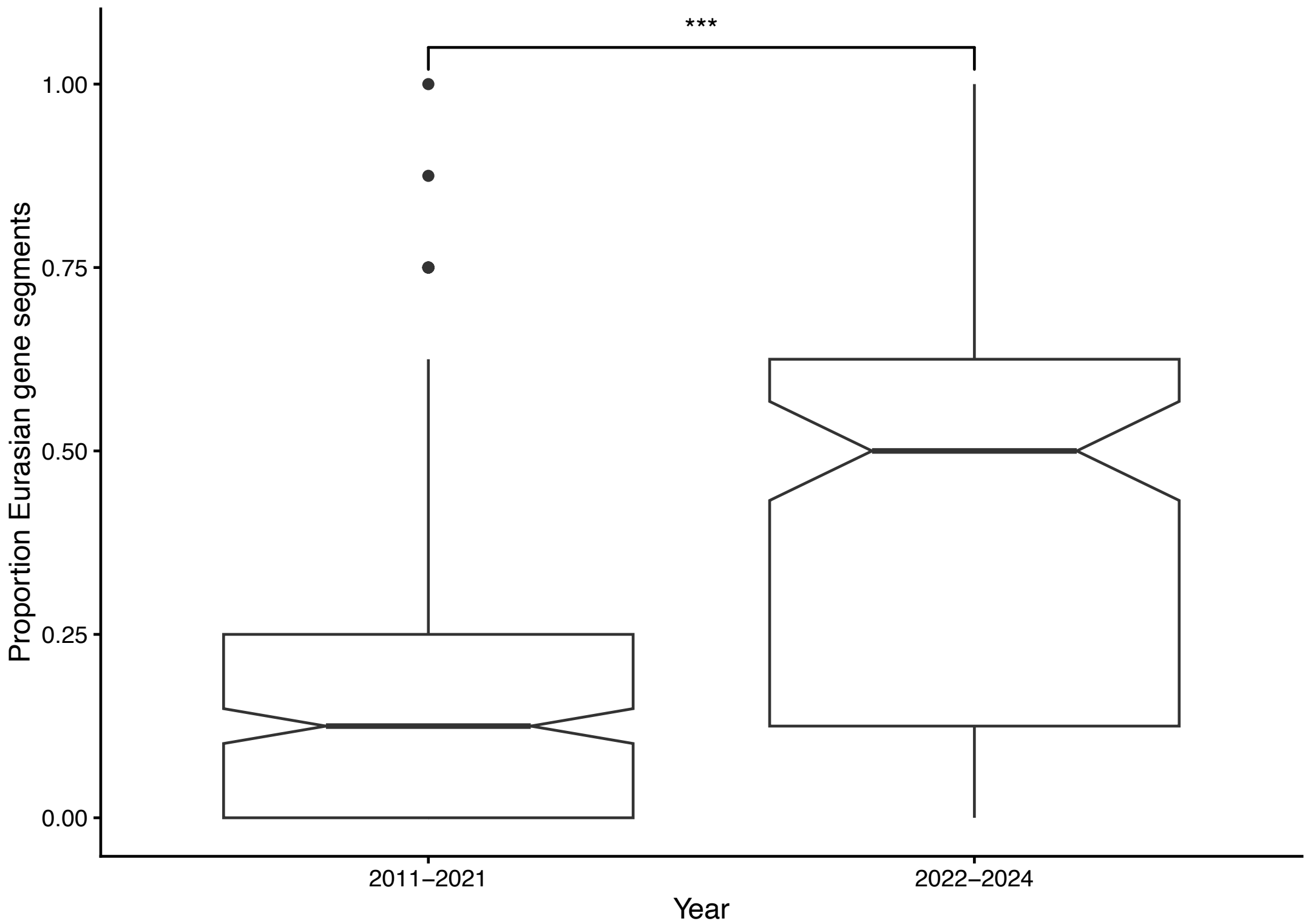

Supplementary Figure 4 Boxplot indicating the proportion of Eurasian-lineage gene segments among avian influenza viruses recovered prior to highly pathogenic avian influenza (HPAI) virus detection (2011-2021) and following HPAI virus detection (2022-2024) in southwestern Alaska. The lower and upper hinges reflect the first and third quartiles, whiskers extend from the highest and lowest value no further than 1.5 inter-quartile range, dots indicate outliers, and notches estimate a 95% confidence interval around the median values (horizontal line). Statistical significance is indicated by asterisks ($p \leq 0.001$).

Supplementary Table 1 Number of avian influenza virus subtype combinations detected in different functional groups.

Subtype	Dabbling duck	Diving or sea duck	Goose	Gull	Total
H1N1	6		3	1	10
H1N2	4				4
H1N3	1				1
H2N3	2		1		3
H2N9	1		1		2
H3N1	1				1
H3N2	7				7
H3N3	1				1
H3N6	11				11
H3N8	104	2	12	3	121
H4N1	1				1
H4N2	6				6
H4N6	37	1	4	2	44
H4N7				1	1
H4N8		1			1
H5N1	71		4		75
H5N2	36		6	3	45
H5N3	1		2		3
H6N1	10				10
H6N2	11				11
H6N5	3		1		4
H6N6	1				1
H6N8	5				5
H7N3	2		4		6
H7N4	3	1			4
H8N4	6				6
H8N8	1				1
H9N1				1	1
H9N2	2		2	1	5
H10N2	1				1
H10N4	1				1
H10N7	12		1		13
H11N2	3		1	2	6

H11N9	2				2
H12N2	1				1
H12N5	1				1
H13N2				10	10
H13N6				1	1
H13N8				1	1
H16N3	3			7	10
mixed	36	1	3	2	42

Supplementary Figure 5 Maximum likelihood phylogenetic trees of individual avian influenza gene segments 1-8. Low pathogenicity avian influenza viruses are annotated with a green circle and highly pathogenic avian influenza (HPAI) viruses are annotated with an orange circle. HPAI genotypes and continental affiliation are indicated to the right of their respective clades. Bootstrap support >70% are indicated at nodes.

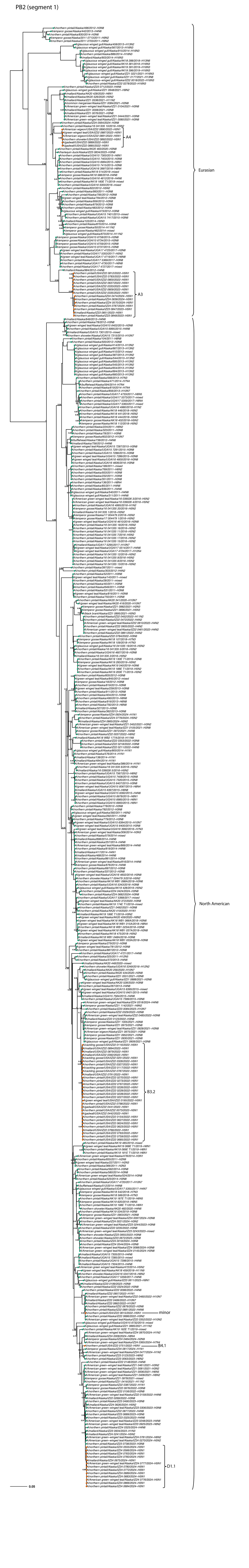

PB1 (segment 2)

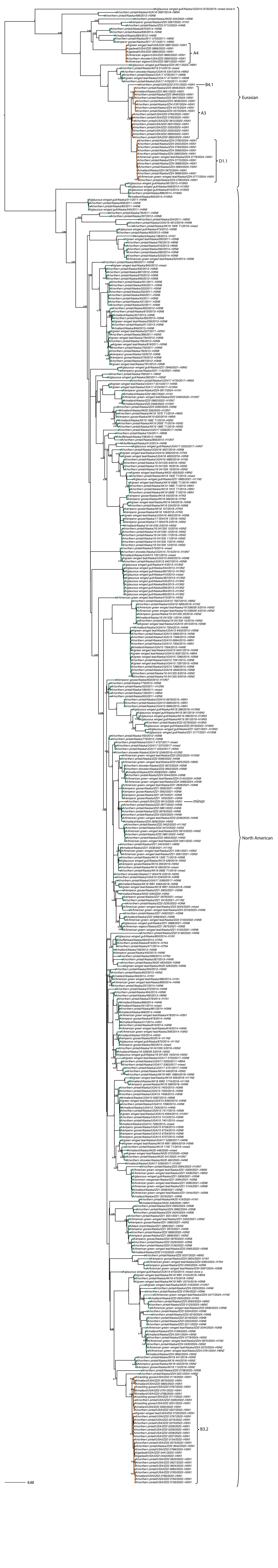

PA (segment 3)

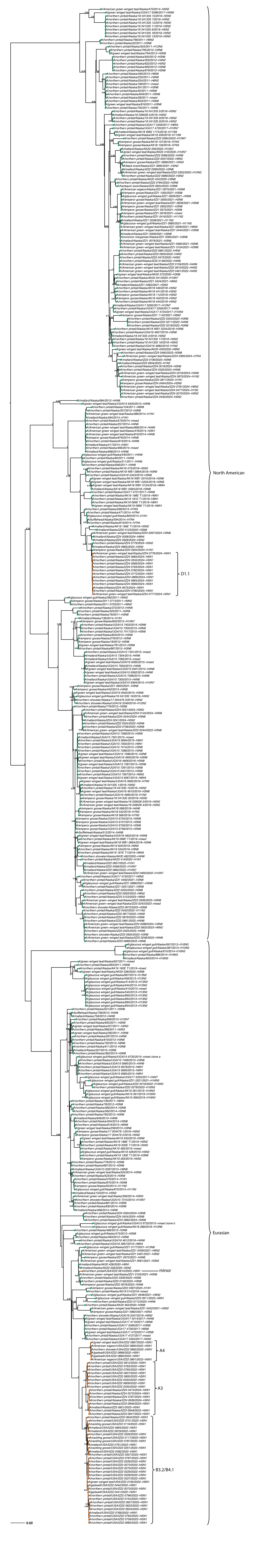

H5 (segment 4)

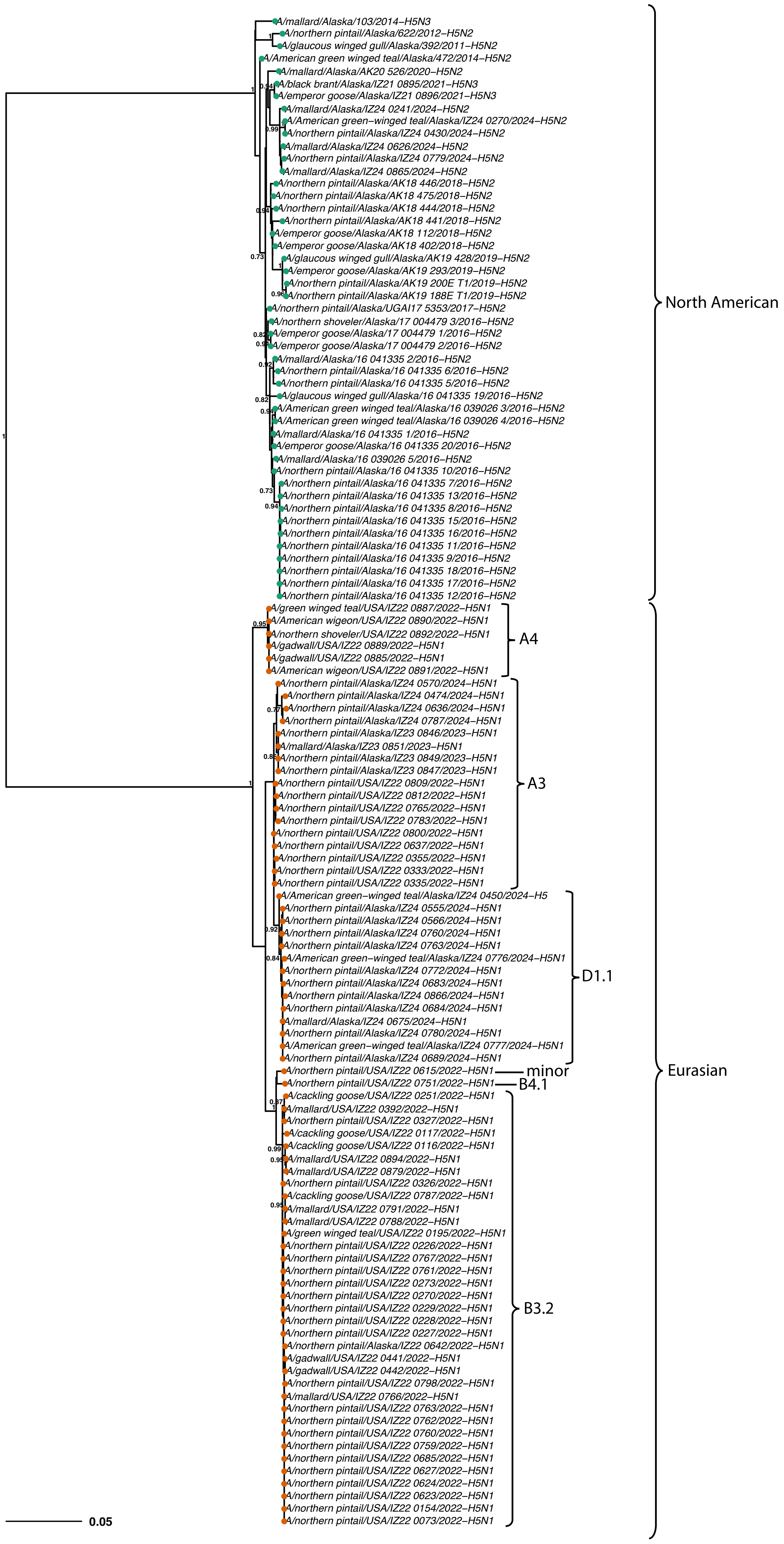
A/mallard/Alaska/103/2014–H5N3
A/northern pintail/Alaska/622/2012–H5N2
A/glaucous winged gull/Alaska/392/2011–H5N2
A/American green winged teal/Alaska/472/2014–H5N2
A/mallard/Alaska/AK20 526/2020–H5N2
A/black brant/Alaska/IZ21 0895/2021–H5N3
A/emperor goose/Alaska/IZ21 0896/2021–H5N3
A/mallard/Alaska/IZ24 0241/2024–H5N2
A/American green–winged teal/Alaska/IZ24 0270/2024–H5N2
A/northern pintail/Alaska/IZ24 0430/2024–H5N2
A/mallard/Alaska/IZ24 0626/2024–H5N2
A/northern pintail/Alaska/IZ24 0779/2024–H5N2
A/mallard/Alaska/IZ24 0865/2024–H5N2
A/northern pintail/Alaska/AK18 446/2018–H5N2
A/northern pintail/Alaska/AK18 475/2018–H5N2
A/northern pintail/Alaska/AK18 444/2018–H5N2
A/northern pintail/Alaska/AK18 441/2018–H5N2
A/emperor goose/Alaska/AK18 112/2018–H5N2
A/emperor goose/Alaska/AK18 402/2018–H5N2
A/glaucous winged gull/Alaska/AK19 428/2019–H5N2
A/emperor goose/Alaska/AK19 293/2019–H5N2
A/northern pintail/Alaska/AK19 200E T1/2019–H5N2
A/northern pintail/Alaska/AK19 188E T1/2019–H5N2
A/northern pintail/Alaska/UGAI17 5353/2017–H5N2
A/northern shoveler/Alaska/17 004479 3/2016–H5N2
A/emperor goose/Alaska/17 004479 1/2016–H5N2
A/emperor goose/Alaska/17 004479 2/2016–H5N2
A/mallard/Alaska/16 041335 2/2016–H5N2
A/northern pintail/Alaska/16 041335 6/2016–H5N2
A/northern pintail/Alaska/16 041335 5/2016–H5N2
A/glaucous winged gull/Alaska/16 041335 19/2016–H5N2
A/American green winged teal/Alaska/16 039026 3/2016–H5N2
A/American green winged teal/Alaska/16 039026 4/2016–H5N2
A/mallard/Alaska/16 041335 1/2016–H5N2
A/emperor goose/Alaska/16 041335 20/2016–H5N2
A/mallard/Alaska/16 039026 5/2016–H5N2
A/northern pintail/Alaska/16 041335 10/2016–H5N2
A/northern pintail/Alaska/16 041335 7/2016–H5N2
A/northern pintail/Alaska/16 041335 13/2016–H5N2
A/northern pintail/Alaska/16 041335 8/2016–H5N2
A/northern pintail/Alaska/16 041335 15/2016–H5N2
A/northern pintail/Alaska/16 041335 16/2016–H5N2
A/northern pintail/Alaska/16 041335 11/2016–H5N2
A/northern pintail/Alaska/16 041335 9/2016–H5N2
A/northern pintail/Alaska/16 041335 18/2016–H5N2
A/northern pintail/Alaska/16 041335 17/2016–H5N2
A/northern pintail/Alaska/16 041335 12/2016–H5N2
North American
A/green winged teal/USA/IZ22 0887/2022–H5N1
A/American wigeon/USA/IZ22 0890/2022–H5N1
A/northern shoveler/USA/IZ22 0892/2022–H5N1
A/gadwall/USA/IZ22 0889/2022–H5N1
A/gadwall/USA/IZ22 0885/2022–H5N1
A/American wigeon/USA/IZ22 0891/2022–H5N1
A4
A/northern pintail/Alaska/IZ24 0570/2024–H5N1
A/northern pintail/Alaska/IZ24 0474/2024–H5N1
A/northern pintail/Alaska/IZ24 0636/2024–H5N1
A/northern pintail/Alaska/IZ24 0787/2024–H5N1
A/northern pintail/Alaska/IZ23 0846/2023–H5N1
A/mallard/Alaska/IZ23 0851/2023–H5N1
A/northern pintail/Alaska/IZ23 0849/2023–H5N1
A/northern pintail/Alaska/IZ23 0847/2023–H5N1
A/northern pintail/USA/IZ22 0809/2022–H5N1
A/northern pintail/USA/IZ22 0812/2022–H5N1
A/northern pintail/USA/IZ22 0765/2022–H5N1
A/northern pintail/USA/IZ22 0783/2022–H5N1
A/northern pintail/USA/IZ22 0800/2022–H5N1
A/northern pintail/USA/IZ22 0637/2022–H5N1
A/northern pintail/USA/IZ22 0355/2022–H5N1
A/northern pintail/USA/IZ22 0333/2022–H5N1
A/northern pintail/USA/IZ22 0335/2022–H5N1
A3
A/American green–winged teal/Alaska/IZ24 0450/2024–H5
A/northern pintail/Alaska/IZ24 0555/2024–H5N1
A/northern pintail/Alaska/IZ24 0566/2024–H5N1
A/northern pintail/Alaska/IZ24 0760/2024–H5N1
A/northern pintail/Alaska/IZ24 0763/2024–H5N1
A/American green–winged teal/Alaska/IZ24 0776/2024–H5N1
A/northern pintail/Alaska/IZ24 0772/2024–H5N1
A/northern pintail/Alaska/IZ24 0683/2024–H5N1
A/northern pintail/Alaska/IZ24 0866/2024–H5N1
A/northern pintail/Alaska/IZ24 0684/2024–H5N1
A/mallard/Alaska/IZ24 0675/2024–H5N1
A/northern pintail/Alaska/IZ24 0780/2024–H5N1
A/American green–winged teal/Alaska/IZ24 0777/2024–H5N1
A/northern pintail/Alaska/IZ24 0689/2024–H5N1
D1.1
A/northern pintail/USA/IZ22 0615/2022–H5N1
minor
A/northern pintail/USA/IZ22 0751/2022–H5N1
B4.1
A/cackling goose/USA/IZ22 0251/2022–H5N1
A/mallard/USA/IZ22 0392/2022–H5N1
A/northern pintail/USA/IZ22 0327/2022–H5N1
A/cackling goose/USA/IZ22 0117/2022–H5N1
A/cackling goose/USA/IZ22 0116/2022–H5N1
A/mallard/USA/IZ22 0894/2022–H5N1
A/mallard/USA/IZ22 0879/2022–H5N1
A/northern pintail/USA/IZ22 0326/2022–H5N1
A/cackling goose/USA/IZ22 0787/2022–H5N1
A/mallard/USA/IZ22 0791/2022–H5N1
A/mallard/USA/IZ22 0788/2022–H5N1
A/green winged teal/USA/IZ22 0195/2022–H5N1
A/northern pintail/USA/IZ22 0226/2022–H5N1
A/northern pintail/USA/IZ22 0767/2022–H5N1
A/northern pintail/USA/IZ22 0761/2022–H5N1
A/northern pintail/USA/IZ22 0273/2022–H5N1
A/northern pintail/USA/IZ22 0270/2022–H5N1
A/northern pintail/USA/IZ22 0229/2022–H5N1
A/northern pintail/USA/IZ22 0228/2022–H5N1
A/northern pintail/USA/IZ22 0227/2022–H5N1
A/northern pintail/Alaska/IZ22 0642/2022–H5N1
A/gadwall/USA/IZ22 0441/2022–H5N1
A/gadwall/USA/IZ22 0442/2022–H5N1
A/northern pintail/USA/IZ22 0798/2022–H5N1
A/mallard/USA/IZ22 0766/2022–H5N1
A/northern pintail/USA/IZ22 0763/2022–H5N1
A/northern pintail/USA/IZ22 0762/2022–H5N1
A/northern pintail/USA/IZ22 0760/2022–H5N1
A/northern pintail/USA/IZ22 0759/2022–H5N1
A/northern pintail/USA/IZ22 0685/2022–H5N1
A/northern pintail/USA/IZ22 0627/2022–H5N1
A/northern pintail/USA/IZ22 0624/2022–H5N1
A/northern pintail/USA/IZ22 0623/2022–H5N1
A/northern pintail/USA/IZ22 0154/2022–H5N1
A/northern pintail/USA/IZ22 0073/2022–H5N1
B3.2
Eurasian
0.05

NP (segment 5)

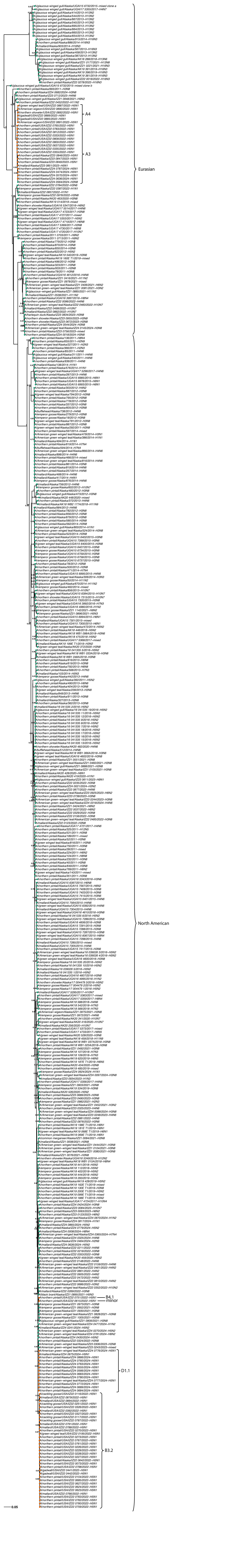

N1 (segment 6)

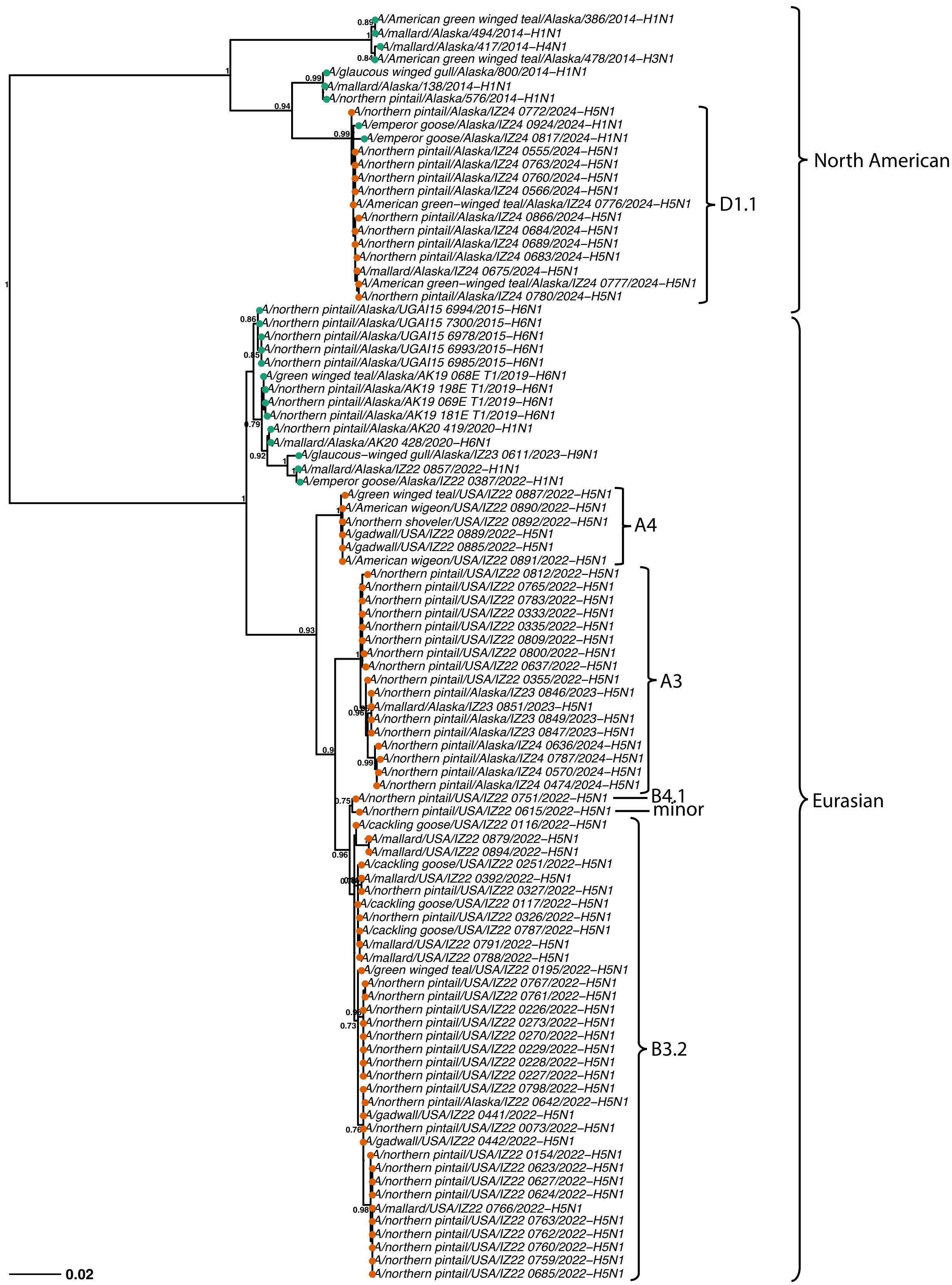
A/American green winged teal/Alaska/386/2014–H1N1
A/mallard/Alaska/494/2014–H1N1
A/mallard/Alaska/417/2014–H4N1
A/American green winged teal/Alaska/478/2014–H3N1
A/glaucous winged gull/Alaska/800/2014–H1N1
A/mallard/Alaska/138/2014–H1N1
A/northern pintail/Alaska/576/2014–H1N1
A/northern pintail/Alaska/IZ24 0772/2024–H5N1
A/emperor goose/Alaska/IZ24 0924/2024–H1N1
A/emperor goose/Alaska/IZ24 0817/2024–H1N1
A/northern pintail/Alaska/IZ24 0555/2024–H5N1
A/northern pintail/Alaska/IZ24 0763/2024–H5N1
A/northern pintail/Alaska/IZ24 0760/2024–H5N1
A/northern pintail/Alaska/IZ24 0566/2024–H5N1
A/American green–winged teal/Alaska/IZ24 0776/2024–H5N1
A/northern pintail/Alaska/IZ24 0866/2024–H5N1
A/northern pintail/Alaska/IZ24 0684/2024–H5N1
A/northern pintail/Alaska/IZ24 0689/2024–H5N1
A/northern pintail/Alaska/IZ24 0683/2024–H5N1
A/mallard/Alaska/IZ24 0675/2024–H5N1
A/American green–winged teal/Alaska/IZ24 0777/2024–H5N1
A/northern pintail/Alaska/IZ24 0780/2024–H5N1
D1.1
North American
A/northern pintail/Alaska/UGAI15 6994/2015–H6N1
A/northern pintail/Alaska/UGAI15 7300/2015–H6N1
A/northern pintail/Alaska/UGAI15 6978/2015–H6N1
A/northern pintail/Alaska/UGAI15 6993/2015–H6N1
A/northern pintail/Alaska/UGAI15 6985/2015–H6N1
A/green winged teal/Alaska/AK19 068E T1/2019–H6N1
A/northern pintail/Alaska/AK19 198E T1/2019–H6N1
A/northern pintail/Alaska/AK19 069E T1/2019–H6N1
A/northern pintail/Alaska/AK19 181E T1/2019–H6N1
A/northern pintail/Alaska/AK20 419/2020–H1N1
A/mallard/Alaska/AK20 428/2020–H6N1
A/glaucous–winged gull/Alaska/IZ23 0611/2023–H9N1
A/mallard/Alaska/IZ22 0857/2022–H1N1
A/emperor goose/Alaska/IZ22 0387/2022–H1N1
A/green winged teal/USA/IZ22 0887/2022–H5N1
A/American wigeon/USA/IZ22 0890/2022–H5N1
A/northern shoveler/USA/IZ22 0892/2022–H5N1
A/gadwall/USA/IZ22 0889/2022–H5N1
A/gadwall/USA/IZ22 0885/2022–H5N1
A/American wigeon/USA/IZ22 0891/2022–H5N1
A4
A/northern pintail/USA/IZ22 0812/2022–H5N1
A/northern pintail/USA/IZ22 0765/2022–H5N1
A/northern pintail/USA/IZ22 0783/2022–H5N1
A/northern pintail/USA/IZ22 0333/2022–H5N1
A/northern pintail/USA/IZ22 0335/2022–H5N1
A/northern pintail/USA/IZ22 0809/2022–H5N1
A/northern pintail/USA/IZ22 0800/2022–H5N1
A/northern pintail/USA/IZ22 0637/2022–H5N1
A/northern pintail/USA/IZ22 0355/2022–H5N1
A/northern pintail/Alaska/IZ23 0846/2023–H5N1
A/mallard/Alaska/IZ23 0851/2023–H5N1
A/northern pintail/Alaska/IZ23 0849/2023–H5N1
A/northern pintail/Alaska/IZ23 0847/2023–H5N1
A/northern pintail/Alaska/IZ24 0636/2024–H5N1
A/northern pintail/Alaska/IZ24 0787/2024–H5N1
A/northern pintail/Alaska/IZ24 0570/2024–H5N1
A/northern pintail/Alaska/IZ24 0474/2024–H5N1
A3
A/northern pintail/USA/IZ22 0751/2022–H5N1
B4.1
A/northern pintail/USA/IZ22 0615/2022–H5N1
minor
A/cackling goose/USA/IZ22 0116/2022–H5N1
A/mallard/USA/IZ22 0879/2022–H5N1
A/mallard/USA/IZ22 0894/2022–H5N1
A/cackling goose/USA/IZ22 0251/2022–H5N1
A/mallard/USA/IZ22 0392/2022–H5N1
A/northern pintail/USA/IZ22 0327/2022–H5N1
A/cackling goose/USA/IZ22 0117/2022–H5N1
A/northern pintail/USA/IZ22 0326/2022–H5N1
A/cackling goose/USA/IZ22 0787/2022–H5N1
A/mallard/USA/IZ22 0791/2022–H5N1
A/mallard/USA/IZ22 0788/2022–H5N1
A/green winged teal/USA/IZ22 0195/2022–H5N1
A/northern pintail/USA/IZ22 0767/2022–H5N1
A/northern pintail/USA/IZ22 0761/2022–H5N1
A/northern pintail/USA/IZ22 0226/2022–H5N1
A/northern pintail/USA/IZ22 0273/2022–H5N1
A/northern pintail/USA/IZ22 0270/2022–H5N1
A/northern pintail/USA/IZ22 0229/2022–H5N1
A/northern pintail/USA/IZ22 0228/2022–H5N1
A/northern pintail/USA/IZ22 0227/2022–H5N1
A/northern pintail/USA/IZ22 0798/2022–H5N1
A/northern pintail/Alaska/IZ22 0642/2022–H5N1
A/gadwall/USA/IZ22 0441/2022–H5N1
A/northern pintail/USA/IZ22 0073/2022–H5N1
A/gadwall/USA/IZ22 0442/2022–H5N1
A/northern pintail/USA/IZ22 0154/2022–H5N1
A/northern pintail/USA/IZ22 0623/2022–H5N1
A/northern pintail/USA/IZ22 0627/2022–H5N1
A/northern pintail/USA/IZ22 0624/2022–H5N1
A/mallard/USA/IZ22 0766/2022–H5N1
A/northern pintail/USA/IZ22 0763/2022–H5N1
A/northern pintail/USA/IZ22 0762/2022–H5N1
A/northern pintail/USA/IZ22 0760/2022–H5N1
A/northern pintail/USA/IZ22 0759/2022–H5N1
A/northern pintail/USA/IZ22 0685/2022–H5N1
B3.2
Eurasian
0.02

M (segment 7)

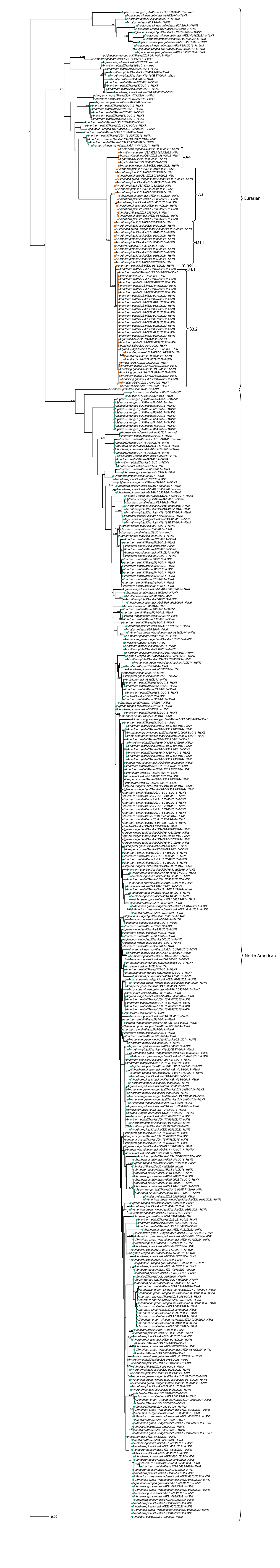

N5 (segment 8)

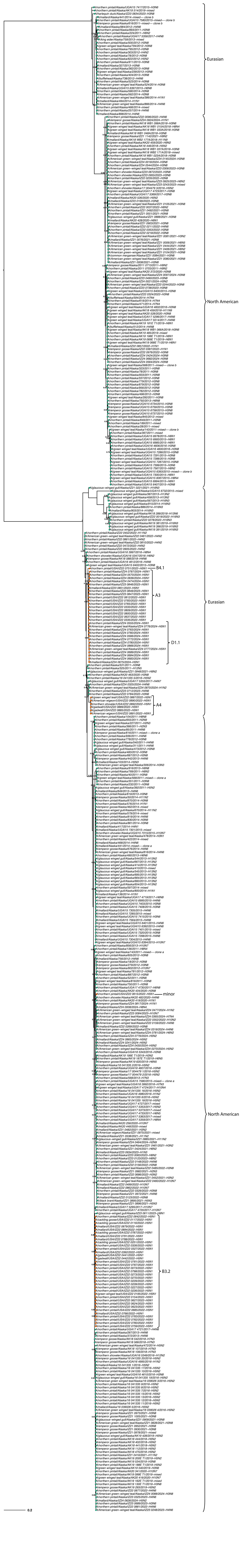
Eurasian
North American
B4.1
A3
Eurasian
D1.1
A4
minor
North American
B3.2
0.2
